# A Manual for the Practice of Surgery

**Published:** 1885-09

**Authors:** 


A Manual for the Practice of Surgery, THOMAS BRYANT, F. R. C. S., Senior Surgeon to and Lecturer on Surgery at Guy's Hospital, London. With seven hundred and twenty-seven illustrations. Fourth edition ; thoroughly revised. Philadelphia: Henry C. Lea's Son & Co. 1885.

American surgeons have unmistakably signified their appreciation of Bryant's Surgery, by demanding four editions since 1876. The present edition is a reprint of the last English edition, which appears in two volumes, but the American publishers have succeeded in presenting the entire work in one volume, a change which will commend itself to all readers. We find some alterations in the substance as well as in the arrangement of the work, as well as some entirely new chapters, and an increase in the number of illustrations, but every change has been for the better, and the present volume will sustain its reputation as the best epitome of the main principles and methods of practice within the reach of the practitioner or student. The publishers have issued it in a neat and attractive form, and although the work contains 1040 pages, it is not inconveniently large.

